# How should we monitor the cardiovascular benefit of sodium–glucose cotransporter 2 inhibition?

**DOI:** 10.1186/s12933-020-01191-5

**Published:** 2020-12-07

**Authors:** Atsushi Tanaka, Koichi Node

**Affiliations:** Department of Cardiovascular Medicine, Saga University, 5-1-1 Nabeshima, Saga, 849-8501 Japan

**Keywords:** Sodium glucose co-transporter 2 inhibitor, Heart failure, Cardiovascular benefit, Biomarker

## Abstract

Sodium–glucose cotransporter 2 (SGLT2) inhibitors are increasingly prescribed for the treatment of patients with type 2 diabetes to reduce the risk of cardiovascular events, including heart failure (HF). The mechanisms by which SGLT2 inhibitors reduce such risk are likely to be independent of diabetes status and improvement of glycemic control. In this commentary, based on recent mediation analyses of cardiovascular outcome trials with SGLT2 inhibitors, we discuss the prognostic role of a well-known HF-related biomarker, amino-terminal pro-B-type natriuretic peptide (NT-proBNP), in patients receiving SGLT2 inhibitors. Interestingly, the NT-proBNP concentration had a relatively small impact on the SGLT2 inhibitor-associated benefit on HF events, suggesting a limited value in measuring NT-proBNP concentrations to monitor effects on cardiovascular outcomes after initiation of SGLT2 inhibitor therapy. Instead, clinical factors, such as body weight and volume status, were prognostic for cardiovascular outcomes. As shown in some biomarker studies, short-term SGLT2 inhibitor treatment significantly improved volume and HF-related health status, despite the absence of a significant change in NT-proBNP concentration. Given the early and continuous risk reduction in HF events seen in the cardiovascular outcome trials with SGLT2 inhibitors, changes in these fundamental clinical parameters after initiation of SGLT2 inhibitor therapy, independent of NT-proBNP, could be more prognostic and could represent key determinants to identify responders or non-responders to SGLT2 inhibitors for cardiovascular outcomes. Thus, this commentary highlights the clinical importance of establishing how clinicians should monitor patients initiating SGLT2 inhibitor therapy to predict the expected cardiovascular benefit. Further detailed investigations and discussion to better understand this ‘‘black box’’ are urgently warranted.

Sodium–glucose cotransporter 2 (SGLT2) inhibitors have a protective effect on the cardiovascular system beyond their glucose-lowering effect [1] and are increasingly prescribed for the treatment of patients with type 2 diabetes (T2D) to reduce the risk of cardiovascular events, including heart failure (HF) [2, 3]. Although the magnitude of the treatment effect of SGLT2 inhibitors on such cardiorenal outcomes varied among the large-scale outcome trials, no explanation for the statistical evidence of heterogeneity in the treatment effects on such outcomes could be clearly identified [4]. This suggests that SGLT2 inhibitors have plausible class effects on cardiorenal outcomes [5, 6]. Indeed, some large-scale observational cohort studies also demonstrated that initiation of SGLT2 inhibitors compared with other glucose-lowering drugs, such as dipeptidyl peptidase-4 inhibitors, was associated with a decreased risk of cardiorenal events among patients with T2D in clinical practice [7–10].

Importantly, results of recent studies indicated that the mechanisms by which SGLT2 inhibitors reduce the risk of adverse cardiorenal events are likely to be independent of diabetes status and improvement of glycemic control [11], and baseline renal filtration function and degree of albuminuria were the most significant indicators of risk for those events [5]. However, it is still uncertain how clinicians should monitor patients who have started SGLT2 inhibitor therapy to predict the expected cardiovascular benefit (Fig. 1).

**Fig. 1:**
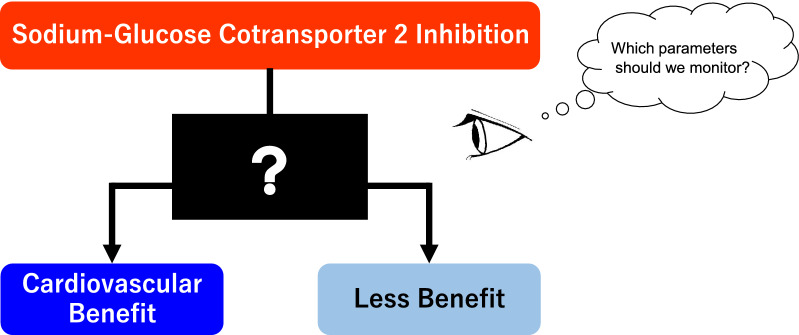
How and what should we monitor to predict the cardiovascular benefit of sodium–glucose cotransporter 2 inhibitors?

Amino-terminal pro-B-type natriuretic peptide (NT-proBNP) is an established biomarker that is useful in the diagnosis of HF and can predict the risk for adverse cardiovascular events [12]. Furthermore, natriuretic peptide-guided treatment is known to be able to improve clinical outcomes and reduce HF-related events irrespective of history of HF [13, 14]. Recently, Januzzi et al. [15] reported concentrations of NT-proBNP over six years using data obtained from the CANVAS program and found that a substantial proportion of patients had elevated levels of NT-proBNP, irrespective of prior history of HF, contributing to a greater risk of cardiovascular events. In addition, canagliflozin, relative to placebo, attenuated the rise in NT-proBNP concentrations over time, and this was consistent with results of a previous study in older adults with T2D [16]. These findings suggest that the reduction in NT-proBNP concentrations with canagliflozin was associated with better cardiovascular outcomes. However, a mediation analysis demonstrated that NT-proBNP lowering had a relatively small effect on the canagliflozin-associated benefit on HF events, suggesting a limited value in measuring NT-proBNP concentrations to monitor effects on cardiovascular outcomes after initiation of SGLT2 inhibitor therapy. Regarding potential mediators associated with improvement of outcomes in another clinical trial with SGLT2 inhibitor therapy, a previous mediation analysis of the EMPA-REG OUTCOME trial showed that hematocrit and hemoglobin, indicative of a hemodynamic effect, were the most important mediators of the reduction in the risk of cardiovascular death [17].

Interestingly, recent small studies investigating clinical surrogate markers, including NT-proBNP, in patients with established HF showed that short-term SGLT2 inhibitor intervention did not decrease the NT-proBNP level compared with glimepiride [18] or placebo [19]. Instead, a 6-month course of canagliflozin decreased body weight and altered volume status, as assessed by hemoconcentration and plasma volume [18]. Furthermore, a 12-week course of dapagliflozin significantly improved HF-related health status, as assessed by the Kansas City Cardiomyopathy Questionnaire [19]. Similarly, a 6-month course of empagliflozin in patients with T2D and known coronary artery disease was associated with a significant reduction in left ventricular mass, as measured by cardiac magnetic resonance imaging, although no significant effect of empagliflozin, compared to placebo, on NT-proBNP concentration was observed [20].

Given the early and continuous risk reduction in HF events seen in the previous outcomes trials with SGLT2 inhibitors, changes in these clinical parameters after initiation of an SGLT2 inhibitor, independent of NT-proBNP concentration, could be more prognostic and could represent key determinants to identify responders or non-responders to SGLT2 inhibitors for cardiovascular outcomes. Thus, it is urgently required to establish how clinicians should monitor patients who have initiated SGLT2 inhibitor therapy to predict its cardiovascular benefit (Fig. 1).

## Data Availability

Not applicable.
